# Phase I clinical trial of HER2-specific immunotherapy with concomitant HER2 kinase inhibtion

**DOI:** 10.1186/1479-5876-10-28

**Published:** 2012-02-10

**Authors:** Erika Hamilton, Kimberly Blackwell, Amy C Hobeika, Timothy M Clay, Gloria Broadwater, Xiu-Rong Ren, Wei Chen, Henry Castro, Frederic Lehmann, Neil Spector, Junping Wei, Takuya Osada, H Kim Lyerly

**Affiliations:** 1 Department of Medicine, Division of Medical Oncology, Duke University Medical Center, Durham, NC, USA; 2 Department of Surgery, Division of General Surgery, Duke University Medical Center, Durham, NC, USA; 3 GlaxoSmithKline Biologicals, Rixensart, Belgium; 4 Cancer Statistical Center, Duke Cancer Institute, Durham, NC, USA; 5 Department of Medicine, Division of Gastroenterology, Duke University Medical Center, Durham, NC, USA; 6 Department of Surgery, Division of Surgical Sciences, Duke University Medical Center, Durham, NC, USA

**Keywords:** HER2, Antitumor immunity, Immunization, Breast cancer

## Abstract

**Background:**

Patients with HER2-overexpressing metastatic breast cancer, despite initially benefiting from the monoclonal antibody trastuzumab and the EGFR/HER2 tyrosine kinase inhibitor lapatinib, will eventually have progressive disease. HER2-based vaccines induce polyclonal antibody responses against HER2 that demonstrate enhanced anti-tumor activity when combined with lapatinib in murine models. We wished to test the clinical safety, immunogenicity, and activity of a HER2-based cancer vaccine, when combined with lapatinib.

**Methods:**

We immunized women (n = 12) with metastatic, trastuzumab-refractory, HER2-overexpressing breast cancer with dHER2, a recombinant protein consisting of extracellular domain (ECD) and a portion of the intracellular domain (ICD) of HER2 combined with the adjuvant AS15, containing MPL, QS21, CpG and liposome. Lapatinib (1250 mg/day) was administered concurrently. Peripheral blood antibody and T cell responses were measured.

**Results:**

This regimen was well tolerated, with no cardiotoxicity. Anti-HER2-specific antibody was induced in all patients whereas HER2-specific T cells were detected in one patient. Preliminary analyses of patient serum demonstrated downstream signaling inhibition in HER2 expressing tumor cells. The median time to progression was 55 days, with the majority of patients progressing prior to induction of peak anti-HER2 immune responses; however, 300-day overall survival was 92% (95% CI: 77-100%).

**Conclusions:**

dHER2 combined with lapatinib was safe and immunogenic with promising long term survival in those with HER2-overexpressing breast cancers refractory to trastuzumab. Further studies to define the anticancer activity of the antibodies induced by HER2 vaccines along with lapatinib are underway.

**Trial registry:**

ClinicalTrials.gov NCT00952692

## Introduction

The human epidermal growth factor receptor 2 (HER2), overexpressed in 20-30% of breast cancers, is associated with more aggressive tumor behavior [1]. Treatment with combinations of the anti-HER2 antibody trastuzumab and chemotherapy lengthens survival in patients with metastatic HER2-overexpressing breast cancer [2]. However, progressive disease typically occurs within one year. Lapatinib, a potent reversible inhibitor of HER2 and epidermal growth factor receptor (EGFR) tyrosine kinases [3], in conjunction with chemotherapy, increases time to progression in these patients [4]. Unfortunately, responses to lapatinib are generally short-lived, and progression remains a significant clinical problem.

Intriguingly, the overexpression of HER2 persists in trastuzumab and lapatinib-refractory tumors [5,6], and thus, targeting HER2 with cancer immunotherapy is a potentially effective strategy. A variety of vaccines targeting HER2, based on proteins, peptides, modified tumor cells, viral vectors, pDNA and dendritic cells (DC) have been developed. Results from phase I and II studies of HER2-targeting cancer vaccines [7] have demonstrated that HER2 is immunogenic, and that immune responses against HER2 may be associated with an improved clinical outcome [8-13].

One protein-based vaccine, dHER2 Antigen-Specific Cancer Immunotherapeutic (ASCI) a recombinant HER2 protein, including a truncated intracellular domain (ICD) and the complete extracellular domain (ECD), combined with the immunological adjuvant AS15, containing MPL, QS21, CpG and liposome, was evaluated in two early phase clinical studies of patients with HER2-overexpressing breast cancer (NCT 00058526 and NCT 00140738) [14]. In both studies, the data showed that dHER2 immunizations were well tolerated, consistently immunogenic at the 500 μg dose and that clinical activity (including prolonged stable disease) was associated with antibody and T cell responses.

One important observation from the prior dHER2 ASCI studies was that the polyclonal antibody-containing serum from immunized patients had functional activity against signaling pathways mediated by HER2. Specifically, incubation of breast cancer cell lines with serum from two immunized patients demonstrated an impact on molecular pathways resembling that of trastuzumab [14]. Because clinical trials have demonstrated that combinations of lapatinib and trastuzumab lead to enhanced clinical activity and combined effects on signaling pathways [15], there has been interest in combining the polyclonal anti-HER2 serum with trastuzumab and indeed, increased apoptosis of human HER2-overexpressing breast cancer cells was observed when lapatinib was combined with HER2-specific polyclonal antisera generated from rabbits immunized with dHER2 ASCI [16]. We therefore hypothesized that the lapatinib would enhance the anti-signaling activity of the polyclonal Abs induced by the dHER2 vaccine in humans. First, it was necessary to establish that the induction of anti-HER2 antibodies by the dHER2 vaccine was not affected by lapatinib and this was the primary purpose of this study.

## Methods

### Patients

Patients provided consent under a protocol approved by the Duke University Medical Center Institutional Review Board. Enrollment requirements were age 18 or older, stage IV HER2- overexpressing (HER2 3+ or FISH +) breast cancer, documented disease progression or relapse following at least one prior standard therapy containing trastuzumab, ECOG status of 0 or 1, adequate hematologic counts, hepatic and renal function and an LVEF of 50% or greater. Concurrent bisphosphonates and hormonal therapy were permitted. Prior chemotherapy and/or trastuzumab were required to have been discontinued no sooner than four and three weeks, respectively, before the first ASCI administration. Initially, prior lapatinib was not permitted; however, this severely limited enrollment and therefore, an amendment was made to permit prior and ongoing lapatinib use. Known autoimmune disease, immunosuppressive therapies or HIV, significant cardiovascular disease or arrhythmias were exclusionary criteria.

### Treatment/Monitoring

**Figure 1:**
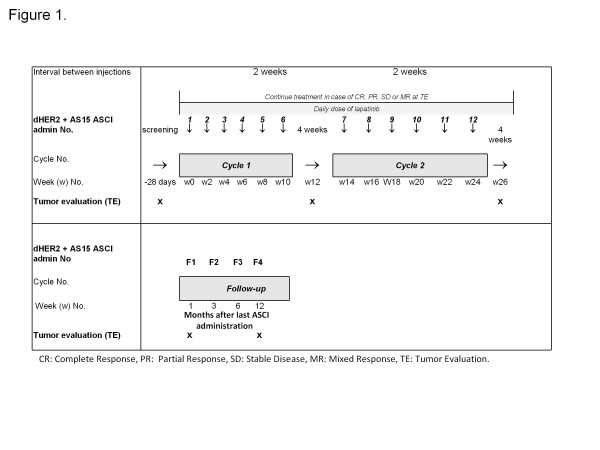
**Study treatment schematic**.

The dHER2 ASCI containing 500 μg of recombinant dHER2 protein (a truncated intra-cellular sequence (ICD) and the complete extracellular sequence (ECD)), reconstituted in the AS15 adjuvant, a liposomal formulation containing MPL, QS21 and CpG, was administered intramuscularly every 2 weeks for 6 administrations (See Figure 1 depicting one cycle). Up to three total cycles of immunization were permitted unless discontinued due to progression of disease. Clinical tumor evaluations were conducted at baseline and at the end of the cycles (e.g., week 12, week 26). Lapatinib (1250 mg) was taken by mouth daily.

Cardiac monitoring consisted of a 12-lead ECG at screening, and at week 12, week 26, and follow-up visit #1 and a MUGA evaluation of ejection fraction (EF) at screening, week 6, 12, 20, 26, follow-up visits #1 and #4.

### Analysis of anti-HER2 antibody binding by ELISA

Serum was collected pre- (week 0) and every 2 weeks while on study. 96-well plates were coated with HER2 ICD protein (5 μg/well) or HER2 ECD protein (5 μg/well) and incubated with 100 μl of serially diluted patient serum or trastuzumab. Plates were then incubated with mouse anti-human IgG conjugated alkaline phosphatase (AP) (Sigma) and developed using p-nitrophenyl phosphate (Sigma). Absorbance (415 nm) was read using a BioRad microplate reader. Titers were defined as the greatest dilution at which the mean absorbance was at least twice negative control.

### ELISPOT analysis

IFN-γ ELISPOT assays (Mabtech Inc., Cincinnati, OH) were performed according to the manufacturer's instructions as previously described 16]. Briefly, PBMC (250,000 cells/well) were added to each well and stimulated overnight with HER2 ICD or HER2 ECD overlapping peptide mixes (1 μg/ml) to detect HER2-specific responses, or CMV peptide mix (2.6 μg/ml), HIV peptide mix (2.6 μg/ml) (both from BD Bioscience) and a mixture of PMA (50 ng/ml) and Ionomycin (1 μg/ml) as controls. Results were normalized to the number of spots per 1 × 10^6 ^PBMC. Based on our previously reported experience [17], a positive response was defined as an increase by ≥ 40 spots from baseline.

### Statistical analyses

Descriptive statistics are presented. Progression-free interval was defined as time from trial enrollment to disease progression or death, whichever came first. Overall survival was defined from time of enrollment until death due to any cause. Progression-free and overall survival was calculated using the Kaplan-Meier product limit method.

## Results

### Patient demographics

Twelve (12) patients were enrolled and received treatment on trial. Demographics are listed for each patient in Table 1. Of note, the patients had substantial metastatic disease burden and had been heavily pretreated, all having progressed on prior trastuzumab and 10/12 having progressed on a lapatinib-containing regimen. Four patients completed one full cycle and one completed two full cycles of immunization.

**Table 1:** Patient characteristics

Pt #	Age	Race	ER or PR +	# prior lines tx	# met sites	Trastuzumab in prior regimen (days since)	Prior Lapatinib	# injections	PFS (d)	OS (d)
001	45	W	Y	4	2	Y (23)	N	4	53	615+

002	50	W	Y	3	3	Y (34)	P	6	82	484

003	48	W	N	3	4	Y (24)	C	6	85	596+

004	53	W	N	6	1	N	C	6	86	549+

005	63	W	Y	2	2	N	C	4	48	540+

006	60	W	N	5	4	Y (37)	C	6	86	531+

007	46	B	N	4	1	Y (33)	P	5	69	486+

008	65	W	N	8	3	Y (27)	C	3	41	456+

012	55	W	N	3	2	N	C	5	69	216

013	65	W	N	4	2	Y (27)	C	4	55	316+

014	56	W	N	3	2	Y (25)	C	12	188	310+

015	46	B	Y	3	4	Y (21)	N	4	69	274+

**Median**	**54**			**3.5**	**2**			**5**	**69**	

1) Prior Lapatinib Use (N = no prior use, P = previous progression on lapatinib, but not on regimen immediately prior, C = continued use, patient was on lapatinib in immediately prior regimen and continued the medication onto trial). 2) OS (overall survival): "+" indicates that the patient was still alive at the last follow-up date

dHER2 ASCI vaccination in combination with lapatinib was well tolerated. The most common adverse events (AE) encountered were "constitutional symptoms" such as musculoskeletal pain (8/12, 66% with 0% grade 3), injection site reactions (6/12, 50% with 0% grade 3), myalgia (5/12, 42% with 0% grade 3) and fatigue (5/12, 42% with 1/12, 9% grade 3 due to progression of disease). There were only two serious adverse events (SAE) on study: one patient experienced chest pain which was determined to be secondary to progression with mediastinal lymphadenopathy and one patient suffered a pulmonary embolism deemed not related to treatment. There was one additional grade 3 AE: epistaxis after a lacrimal duct repair. There were no grade 4 AEs observed.

Cardiac function was closely monitored during this study with two evaluations of left ventricular ejection fraction (LVEF) at the beginning of every cycle. No heart failure or any decrease in left ventricular cardiac ejection fraction ≥ 20% relative to baseline was seen for any patient at any time point on trial.

### Antigen specific T Cell and antibody responses

**Figure 2:**
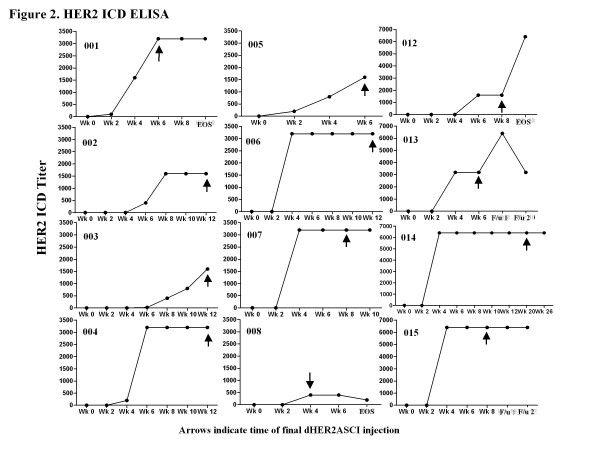
**HER2-ICD specific antibody response**. Serum from patients receiving dHER2-ASCI + lapatinib was analyzed for HER2-ICD specific antibodies by ELISA. Plates were coated with HER2-ICD protein and incubated with serial dilutions of patient sera (1:25-1:6400), along with negative control serum. Individual graphs of the HER2-ICD titer over time are presented for each patient (indicated by study number) with arrows indicating final dHER2ASCI injection.

We analyzed HER2 ICD and ECD specific antibody responses by ELISA. All patients had a detectable anti-ICD-antibody response (ranging from 1:200 to 1:6400) following vaccination in the context of lapatinib treatment. We observed an increase in HER2 ICD antibody titer starting as early as week 4 (after 2 injections; 9 of 12 patients) (Figure 2).

**Figure 3:**
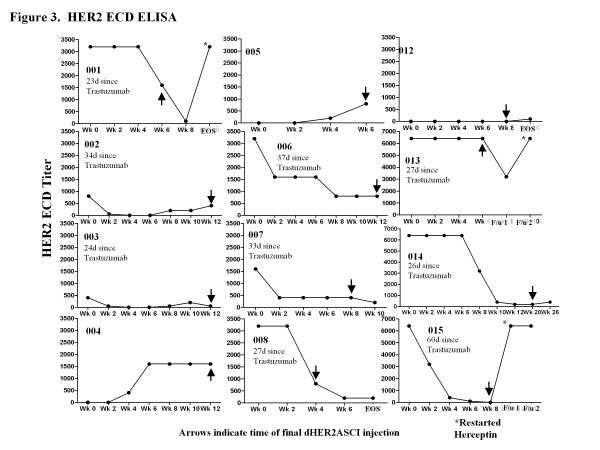
**HER2-ECD specific antibody response**. Serum from patients receiving dHER2-ASCI + lapatinib was analyzed for HER2-ECD specific antibodies by ELISA. Plates were coated with HER2-ECD protein and incubated with serial dilutions of patient sera (1:25- 1:6400), along with negative control serum and Herceptin as a positive control. Individual graphs of the HER2-ECD titer over time is represented for each patient (indicated by study number) with arrows indicating final dHER2ASCI injection. Although all patients had received trastuzumab at some point in the past, nine patients had received trastuzumab with their immediately prior systemic treatment regimen (range 21 to 37 days), while three had received the trastuzumab during a more distant systemic treatment reginmen. The numbers of days between receiving trastuzumab and starting study drug is noted on each graph as applicable. A * indicates samples taken once trastuzumab was restarted by the patient

As expected, detection of dHER2 vaccine-induced ECD-specific antibodies was complicated by detection of pre-vaccination HER2-ECD specific antibodies (due to residual trastuzumab) in the sera of 9 of the 12 patients who had been receiving trastuzumab 23 to 60 days prior to study entry (Figure 3). The 3 patients who did not receive trastuzumab within 2 months of starting the study (patients 4, 5, 12) did not exhibit pre-existing antibodies to HER2-ECD, but had detectable post vaccination HER2-ECD specific responses ranging from 1:100 to 1:1600 (Figure 3). For these 3 patients, the dynamics of the HER2-ICD and -ECD antibody responses over time indicate that HER2-specific antibodies were detectable between 4 to 6 weeks after initiating the vaccinations (i.e., following 3-4 vaccinations) with a greater antibody titer specific for HER2-ICD compared to HER2-ECD (Figures 2 and 3). An additional two patients with detectable pre-vaccination anti-ECD antibodies demonstrated an increase in HER2-ECD antibody titer following vaccination (Figure 3). In the remainder of patients, the pre-vaccination HER2 ECD titers levels were not increased after vaccination, making an accurate assessment of the vaccine induced HER2 ECD-specific response difficult.

HER2 specific T cell responses were analyzed by IFNγ ELISPOT assay on cryopreserved, non-restimulated peripheral blood specimens obtained at each timepoint. There were increments in the ECD-specific T cell precursor frequency in one patient and in the ICD-specific T cell precursor frequency in four patients; however, using a pre-specified definition of a positive response as an increase in 40 spots over pre-vaccination frequency, there was a single patient with an ECD-specific T cell response and none with an ICD-specific T cell response (Additional file 1: Table S1). This patient appears to have had a pre-existing ECD-specific response which was boosted with the vaccine.

### Clinical outcome

**Figure 4:**
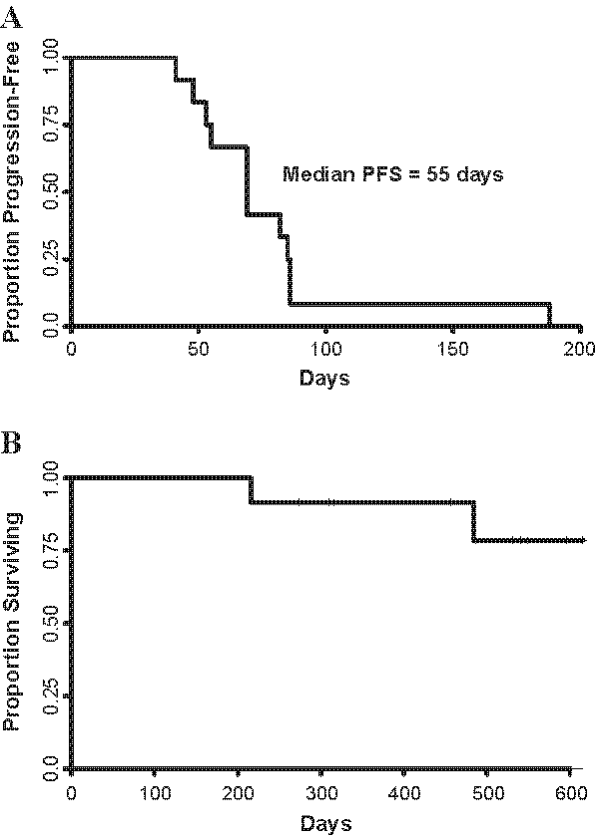
**Outcomes for patients treated with dHER2 ASCI vaccination and lapatinib**. **a**) Kaplan Meier Plot Progression Free Survival **b**) Overall Survival.

There were no objective clinical responses. One patient remained progression free on trial for 6 months. Median time to progression was 55 days (range: 41-188) (Figure 4a). Ten of 12 patients remain alive in follow-up. Overall survival at 300 days was 92% (95% CI: 77-100%) (Figure 4b). Because of the small numbers of patients, we could not statistically compare TTP or overall survival with immune responses; however, there were no obvious correlations.

## Discussion

The purpose of this study was to determine the clinical and immunologic effects of immunization against HER2 concurrent administration of the HER2 tyrosine kinase inhibitor lapatinib. Previously, we reported that a HER2-targeting vaccine could induce antibody and T cell responses when administered concomitantly with lapatinib in a murine model [18]. We extended this work in the present study confirming for the first time in humans that concomitant administration of lapatinib did not appear to affect the immune response induced by the HER2 immunotherapy. Although we are not aware of other data regarding the effect of lapatinib on the immune response in humans, other tyrosine kinase inhibitors have demonstrated negative effects on the immune response, such as sorafenib (which targets RAF protein in the EGFR pathway as well as other targets) while others, such as sunitinib, have had no detrimental effects or potentially improve immune responses [19,20].

We observed potent antibody responses to HER2 ECD and ICD, suggesting that we could still break tolerance to a self-antigen in the context of lapatinib. In the prior phase I/II studies of dHER2 ASCI, antibody responses were consistently observed, and CD4+ and CD8+ T cell responses were also reported. The preferential induction of antibodies using HER2 protein-based vaccines has been already reported. In a series of patients immunized with the truncated 146HER2 protein complexed with cholesteryl pullulan alone or with various adjuvants [21-23], antibody responses against HER2 were detected in 14 of 15 patients, but only 5 out of 9 developed detectable HER2-specific CD8+ and/or CD4+ T-cell immune responses. Because the development of antibodies generally requires Th2 cells, but our method for measuring IFN-gamma ELISPOT identifies Tc1 and Th1, and not Th2 responses, it is possible that a Th2 response occurred, but was not detectable. It is also possible, as has been reported by others using protein-based vaccines, that regulatory T cells (Treg), typically increased in advanced cancer patients, caused decreases in T cell responses [24]. Future strategies to induce greater T cell responses could be warranted as some authors have suggested the T cell responses correlate with clinical outcome [25]. We have developed a series of recombinant viral vectors expressing HER2 that have been evaluated preclinically and were shown to induce HER2-specific CD8+ T cells. These vectors will be entering clinical trials shortly [26]. We have also reported strategies for depleting Treg [27].

One emerging aspect of cancer immunotherapy is that long periods (between 4 to 6 immunizations) are needed to reach an optimal humoral or cellular response. We noted that some patients rapidly progressed during immunization, but prior to development of detectable HER2 specific antibody responses, or until the antibody responses are of sufficient quantity or quality to impact disease. In most cases these were patients who had been maintained on trastuzumab prior to participation. The decline in anti-ECD Ab responses in the early weeks of the study likely represents trastuzumab levels declining. This suggests that for future vaccine studies, patients may need to receive concomitant trastuzumab until they have an adequate induction of antibody responses. Disis *et al*. showed that patients could be vaccinated in the setting of trastuzumab without additional toxicity. The expected immune responses including epitope and antigen spreading were observed [28]. Indeed, in our study, two patients with detectable pre-vaccination anti-ECD antibodies demonstrated an increase in HER2-ECD antibody titer following vaccination (Figure 3), suggesting we could also induce antibody responses against the dHER2 despite the presence of trastuzumab.

Recent clinical trials have established longer survival for patients receiving trastuzumab plus lapatinib, suggesting clinical benefit for antibody plus tyrosine kinase inhibition of HER2. Because this was not a randomized trial, we are not able to determine the clinical benefit of combined dHER2 ASCI plus lapatinib; however, our observed median PFS and overall survival in a heavily pretreated population with multiple sites of metastasis who had progressive disease on trastuzumab and prior lapatinib compares favorably with other clinical data. For example, although there is insufficient data in the literature regarding the outcome of patients refractory to both drugs, among patients who have progressive disease while receiving trastuzumab therapy, overall survival has ranged from 10 - 19 months [29-31]. In the EGF104900 study, which randomized patients who had progressed on trastuzumab to either lapatinib alone or lapatinib in combination with trastuzumab, PFS of 8 weeks and OS of 39 weeks (or 273 days) was reported for the lapatinib only arm [31]. It has also been observed in some immunotherapy studies, that OS is improved despite a lack of effect on PFS suggesting that delayed effects of the vaccine might have led us to prematurely discontinue immunization. Future studies should permit a greater extent of progression (such as 50% growth) in asymptomatic progression as has been suggested in the literature [32].

Another important aspect of the strategy of combining a HER2 immunotherapy with lapatinib is the potential combined inhibition of HER2 signaling. A detailed analysis of effects of anti-HER2 serum by our group is reported elsewhere (Rong et al., manuscript submitted). For the current manuscript, we performed a preliminary analysis in which antibodies, purified from post-vaccination serum from selected patients (P002, P004, and P008), were tested for their ability to reduce phosphorylation of the downstream molecule AKT in the HER2 overexpressing cell line SKBR3. In the patient with the highest titer of ECD among the three (P004), pAKT was decreased by antibodies purified from the post-vaccination serum, providing a suggestion that there are anti-signaling effects of the dHER2-induced antibodies (Additional file 2: Figure S1). Serum from patients in the previous phase I/II studies of dHER2 alone bound HER2-overexpressing breast cancer cell lines and inhibited growth of these cell lines with an effect on molecular pathways resembling that of trastuzumab [14]. We previously reported that the polyclonal sera induced in mice by a HER2 vaccine were superior to monoclonal antibodies in mediating receptor internalization and degradation, resulting in ablation of HER2 signaling over time. Furthermore, there was increased anti-tumor activity when this vaccine was administered concomitantly with lapatinib to mice [18]. These data suggest a potential advantage to our approach. First, the vaccine may provide durable periods of exposure to biologic levels of anti-HER2 antibody, whereas trastuzumab must continue to be administered. Second, the vaccine induces polyclonal antibody responses which could have immunologic functions (such as antibody dependent cellular cytotoxicity) as well as direct functions on HER2 signaling. Recently, it was reported that lapatinib induced HER2 surface expression in HER2-positive breast cancer cell lines, leading to the enhancement of trastuzumab-mediated ADCC [33]. These potential advantages strongly support the further assessment of HER2-targeting immunotherapies with concomitant lapatinib and/or trastuzumab in human clinical trials.

One potential disadvantage of the vaccine strategy is that the antibody titers are lower than those achieved during trastuzumab administration, suggesting future vaccine strategies may need to enhance the titer of antibody induced. Based on the tolerability and safety established in the current study, we will be embarking on a study to evaluate the use of viral vector-based vaccine encoding HER2 along with lapatinib. The potential benefits of such immunotherapeutic strategies over a monoclonal antibody approach (e.g., induction of both T cell and polyclonal antibody responses with multiple mechanisms of action) warrant this testing. More broadly, we believe that targeting receptor molecules using immunotherapy as a mean to perturb signaling offers potential new opportunities to target cancer beyond the conventional lytic killing of tumors by the immune system.

In summary, the data presented is consistent with prior preclinical and clinical observations and supports the expansion of combination studies. However, the limited numbers of patients, the inability to assess clinical responses compared with controls, and the potential effects of waning trastuzumab levels are challenges that would be best addressed in a randomized trial.

## Abbreviations

HER2: human epidermal growth factor receptor 2; EGFR: epidermal growth factor receptor; DC: dendritic cells; ASCI: Antigen-Specific Cancer Immunotherapeutic; ICD: intracellular domain; ECD: extracellular domain; EF: ejection fraction; AP: alkaline phosphatase; AE: adverse events; SAE: serious adverse events; LVEF: left ventricular ejection fraction

## Competing interests

EH, KB, ACH, GB, NS, TO, HKL and MAM received financial support from GSK to support the research described in the manuscript. HC, FL, and TC are employed by and/or stockholders of GSK.

## Authors' contributions

EH participated in the design and coordination of the study, data acquisition and analysis, recruitment of patients and helped draft the manuscript, KB participated in the design of the study, recruitment of patients and helped draft the manuscript, AH participated in the coordination of the study, data acquisition, immune data analysis, and helped draft the manuscript, TM participated in the design and helped draft the manuscript, GB performed statistical analysis, HC participated in the design and helped draft the manuscript, FL participated in the design and helped draft the manuscript, NS participated in the design and helped draft the manuscript, JW participated in the data acquisition and analysis, TO participated in the data acquisition and analysis, HKL participated in the design and helped draft the manuscript. All authors gave final approval of the manuscript for publication.

### Funding

This work was supported by the Department of Defense (HKL; W81XWH-06-1-0585) and GlaxoSmithKline Biologicals.

## Supplementary Material

Additional file 1 **Table S1**. dHER2ASCI ELISpot results: Number of IFNg producing. Click here for file

Additional file 2 **Figure S1**. Reduced phosphorylation of AKT following dHER ASCI immunization. Click here for file
