# Correction: phase 1 clinical trial of HER2-specific immunotherapy with concomitant HER2 kinase inhibtion

**DOI:** 10.1186/1479-5876-11-82

**Published:** 2013-03-27

**Authors:** Erika Hamilton, Kimberly Blackwell, Amy C Hobeika, Timothy M Clay, Gloria Broadwater, Xiu-Rong Ren, Wei Chen, Henry Castro, Frederic Lehmann, Neil Spector, Junping Wei, Takuya Osada, H Kim Lyerly, Michael A Morse

**Affiliations:** 1 Department of Medicine, Division of Medical Oncology, Duke University Medical Center, Durham, NC, USA; 2 Department of Surgery, Division of General Surgery, Duke University Medical Center, Durham, NC, USA; 3 GlaxoSmithKline Biologicals, Rixensart, Belgium; 4 Cancer Statistical Center, Duke Cancer Institute, Durham, NC, USA; 5 Department of Medicine, Division of Gastroenterology, Duke University Medical Center, Durham, NC, USA; 6 Department of Surgery, Division of Surgical Sciences, Duke University Medical Center, Durham, NC, USA

## Corrections

In our original manuscript [1], the corresponding author, Michael A Morse, was missed from the authors’ list. Therefore the correct author list should be:

Erika Hamilton, Kimberly Blackwell, Amy C Hobeika, Timothy M Clay, Gloria Broadwater, Xiu-Rong Ren, Wei Chen, Henry Castro, Frederic Lehmann, Neil Spector, Junping Wei, Takuya Osada, H Kim Lyerly and Michael A Morse.

Also the title of our original manuscript ‘Phase 1 clinical trial of HER2-specific immunotherapy with concomitant HER2 kinase inhibtion’ is also incorrect. The title should read:‘Phase 1 clinical trial of HER2-specific immunotherapy with concomitant HER2 kinase inhibition’.

Journal of Translational Medicine regret any inconvenience that this inaccuracy might have caused.

## Competing interests

The authors declare that they have no competing interests.

## Authors’ contribution

All authors read and approved the final manuscript.
