# Supplementary material for: Phase I clinical trial of HER2-specific immunotherapy with concomitant HER2 kinase inhibtion
Source: J Transl Med. 2012 Feb 10;10:28. doi: 10.1186/1479-5876-10-28 (PMC3306270; doi:10.1186/1479-5876-10-28)
Supplement: Additional file 1 — Table S1. dHER2ASCI ELISpot results: Number of IFNg producing. [file 1479-5876-10-28-S1.PPTX]

## Slide 1
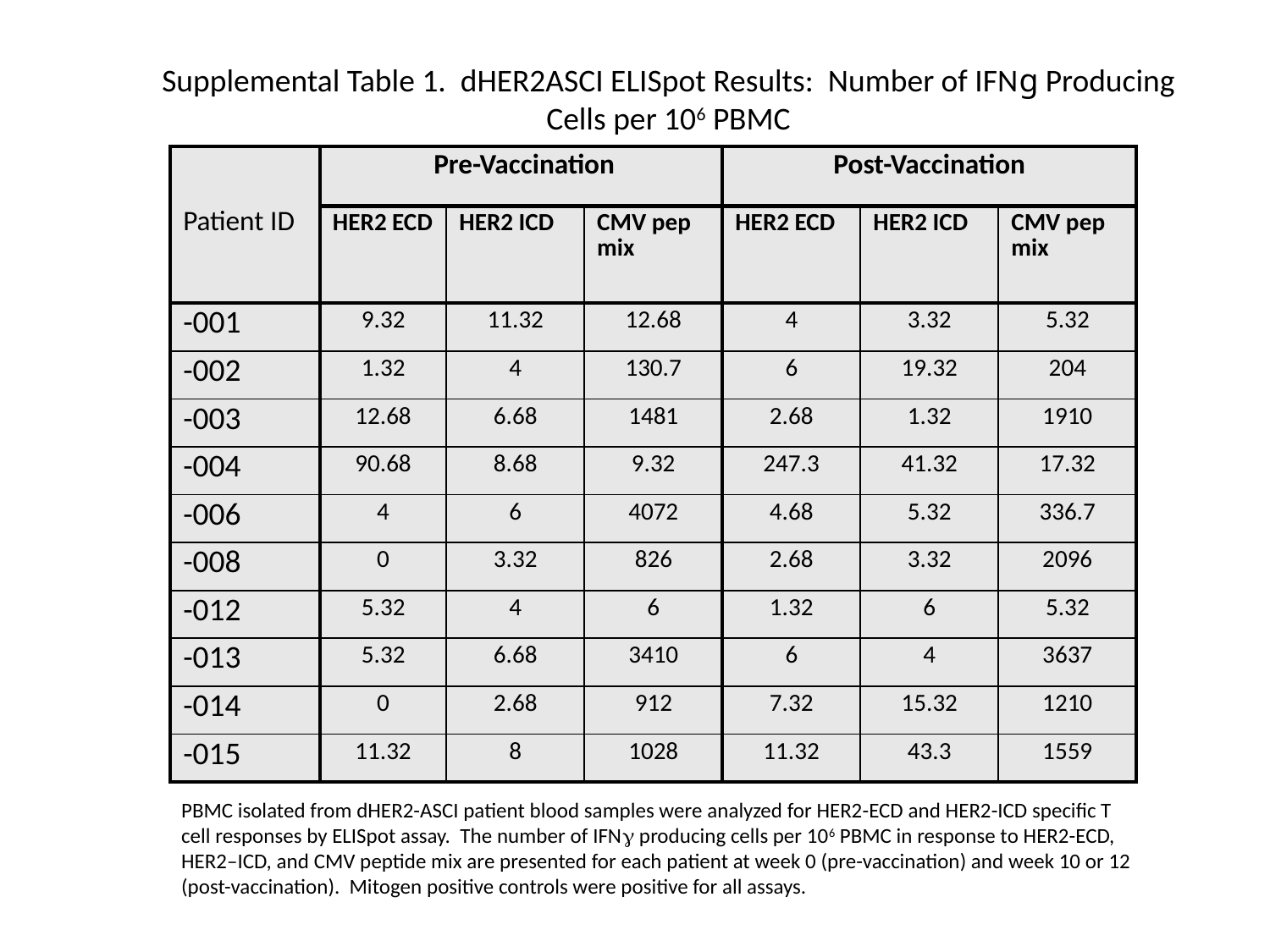

Supplemental Table 1. dHER2ASCI ELISpot Results: Number of IFNg Producing
Cells per 106 PBMC
| Patient ID | Pre-Vaccination | | | Post-Vaccination | | |
| --- | --- | --- | --- | --- | --- | --- |
| | HER2 ECD | HER2 ICD | CMV pep mix | HER2 ECD | HER2 ICD | CMV pep mix |
| -001 | 9.32 | 11.32 | 12.68 | 4 | 3.32 | 5.32 |
| -002 | 1.32 | 4 | 130.7 | 6 | 19.32 | 204 |
| -003 | 12.68 | 6.68 | 1481 | 2.68 | 1.32 | 1910 |
| -004 | 90.68 | 8.68 | 9.32 | 247.3 | 41.32 | 17.32 |
| -006 | 4 | 6 | 4072 | 4.68 | 5.32 | 336.7 |
| -008 | 0 | 3.32 | 826 | 2.68 | 3.32 | 2096 |
| -012 | 5.32 | 4 | 6 | 1.32 | 6 | 5.32 |
| -013 | 5.32 | 6.68 | 3410 | 6 | 4 | 3637 |
| -014 | 0 | 2.68 | 912 | 7.32 | 15.32 | 1210 |
| -015 | 11.32 | 8 | 1028 | 11.32 | 43.3 | 1559 |
PBMC isolated from dHER2-ASCI patient blood samples were analyzed for HER2-ECD and HER2-ICD specific T cell responses by ELISpot assay. The number of IFNg producing cells per 106 PBMC in response to HER2-ECD, HER2–ICD, and CMV peptide mix are presented for each patient at week 0 (pre-vaccination) and week 10 or 12 (post-vaccination). Mitogen positive controls were positive for all assays.
