# Supplementary material for: Phase I clinical trial of HER2-specific immunotherapy with concomitant HER2 kinase inhibtion
Source: J Transl Med. 2012 Feb 10;10:28. doi: 10.1186/1479-5876-10-28 (PMC3306270; doi:10.1186/1479-5876-10-28)
Supplement: Additional file 2 — Figure S1. Reduced phosphorylation of AKT following dHER ASCI immunization. [file 1479-5876-10-28-S2.PPTX]

## Slide 1
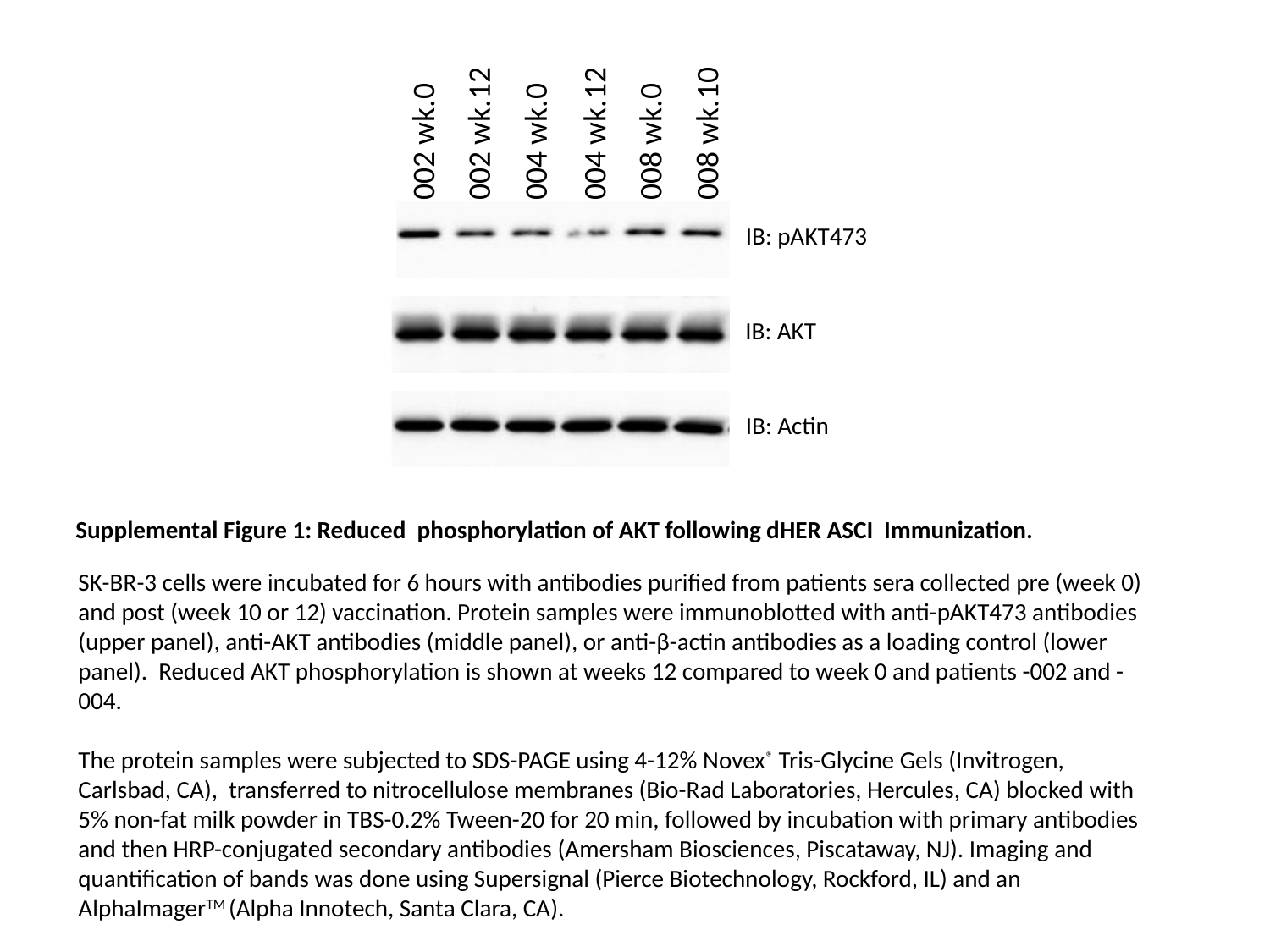

002 wk.12
004 wk.12
008 wk.10
002 wk.0
004 wk.0
008 wk.0
IB: pAKT473
IB: AKT
IB: Actin
Supplemental Figure 1: Reduced phosphorylation of AKT following dHER ASCI Immunization.
SK-BR-3 cells were incubated for 6 hours with antibodies purified from patients sera collected pre (week 0) and post (week 10 or 12) vaccination. Protein samples were immunoblotted with anti-pAKT473 antibodies (upper panel), anti-AKT antibodies (middle panel), or anti-β-actin antibodies as a loading control (lower panel). Reduced AKT phosphorylation is shown at weeks 12 compared to week 0 and patients -002 and -004.
The protein samples were subjected to SDS-PAGE using 4-12% Novex® Tris-Glycine Gels (Invitrogen, Carlsbad, CA), transferred to nitrocellulose membranes (Bio-Rad Laboratories, Hercules, CA) blocked with 5% non-fat milk powder in TBS-0.2% Tween-20 for 20 min, followed by incubation with primary antibodies and then HRP-conjugated secondary antibodies (Amersham Biosciences, Piscataway, NJ). Imaging and quantification of bands was done using Supersignal (Pierce Biotechnology, Rockford, IL) and an AlphaImagerTM (Alpha Innotech, Santa Clara, CA).
